# Study on Seroprevalence and Associated Risk Factors of Newcastle Disease in Smallholder Poultry Farms in Sodo Zuria District, Wolaita Zone, Southern Ethiopia

**DOI:** 10.1155/2022/7478018

**Published:** 2022-09-21

**Authors:** Saliman Aliye, Habtamu Endale, Mesfin Mathewos, Haben Fesseha

**Affiliations:** School of Veterinary Medicine, Wolaita Sodo University, Wolaita Sodo, Ethiopia

## Abstract

Newcastle disease (NCD) is a highly contagious viral disease of poultry and remains a constant threat in poultry farms that causes huge economic losses. The objective of this study was to estimate and assess the seroprevalence and associated risk factors of Newcastle disease in the Sodo Zuria district, southern Ethiopia. A cross-sectional survey followed by a simple random sampling technique was conducted from May to July 2021 on 384 apparently healthy nonvaccinated chickens on 30 smallholder poultry farms using commercial indirect ELISA kits and a questionnaire survey. The data were analyzed by using STATA for windows version 20 and a logistic regression reporting odds ratiowas applied to describe the seroprevalence of Newcastle disease with associated risk factors. The result of the study demonstrates that there was a high seroprevalence 48.7% (*n*= 187/384) of Newcastle disease in the study district. Information on associated risk factors were assessed using a semistructured questionnaire. The sex of the chicken showed a statistically significant difference (*x*^2^ = 4.842; *p* = 0.028) with the seroprevalence of the disease. The difference in seroprevalence among intensive, semi-intensive, and extensive management system was statistically significant (*x*^2^ = 3.84; *p* = 0.0001). There was also a statistical significant difference (*x*^2^ = 2.3854; *p* = 0.496) in the absence and presence of safe disposal of a dead chicken with the occurence of Newcastle disease. However, no statistically significant difference was observed among age groups (*x*^2^ = 4.335; *p* = 0.114), disinfection of poultry house (*x*^2^ = 0.0; *p* = 0.998), presence and absence of footbath (*x*^2^ = 2.969; *p* = 0.085), the breeds (*x*^2^ = 4.490; *p* = 0.106), type of chicken (*x*^2^ = 0.302; *p* = 0.583), and housing system (*x*^2^ = 1.926; *p* = 0.588). A high seroprevalence without vaccination history showed that the virus was circulating within the poultry. Therefore, further molecular study has to be conducted to identify circulating strains and develop an evidence-based control program.

## 1. Introduction

Ethiopia has a huge animal population, with poultry accounting for the majority of it. The country's total chicken population is estimated to be 60.5 million, with 94.33 percent indigenous chickens, 3.21 percent hybrids, and 2.47 percent exotics; the vast majority of these hens (99%) are kept in a traditional system with little or no housing, nutrition, or health care inputs [1].

In favor of Ethiopian agroecology and agronomic practices, as well as a large population of chickens, [2], poultry production in Ethiopia helps underprivileged livestock keepers better their living conditions and prosper economically, contributes to overall agricultural output, and is an important source of animal protein. Poultry (in the form of meat or eggs) contributes significantly to food and nutrition security as well as income for a large portion of the population. The majority of poultry production, particularly in poorer nations, was done in the backyard, primarily to meet household food needs and as a source of additional revenue [3]. The lack of proper scientific information on the incidence and consequences of poultry diseases makes it difficult to create a viable and profitable poultry industry in the country [4].

However, the sector has been adversely affected by a variety of constraints including poor feeding and housing management; the disease is one of the most important constraints to the poultry industry. From this, infectious diseases like Fowl Cholera Newcastle disease (NCD), Infectious Bursal disease (IBD) or Gumboro, and Marek's disease pose a threat to the sector's productivity and vital function. Of these infectious diseases, Newcastle disease is the second leading cause of death in poultry next to fowl cholera [4–6] which is highly contagious and affects various avian species with worldwide distribution and is caused by the single-stranded negative-sense virulent strains of avian orthoavulavirus 1 (AOaV-1; formerly avian paramyxovirus-1; Paramyxoviridae family) [7, 8]. According to [3], the strains of Newcastle can be categorized into five pathotypes: asymptomatic enteric strain, lentogenic strain, mesogenic stain, viscerotropic velogenic strain, and neurotropic velogenic strain based on pathogenicity. NCD disease affects the gastrointestinal, respiratory, and nervous systems of the poultry with a 100% mortality rate.

It is regarded as an important reportable poultry disease and cause of economic loss (through morbidity, mortality, and production decline) in the poultry industry [9–11]. It hinders the global commercial poultry industry by causing significant illness and death in poultry [12, 13].

The productivity and contribution of chicken production to the achievement of Ethiopia's food security [14] are overwhelmed by different constraints including poultry diseases like NCD at Sodo Zuria district of Wolaita Zone [15]. So far, no study was conducted on the seroprevalence and associated risk factors of the Newcastle disease in the study district, and most of the study was done on a questionnaire survey. Hence, it is crucial to study the associated risk factors and the seroprevalence of Newcastle disease in smallholder poultry production farms to have more information to implement appropriate control and prevention strategies. Therefore, the objective of this study was to determine the seroprevalence and assess associated risk factors of Newcastle disease in smallholder poultry farms of Sodo Zuria district of Wolaita zone, southern Ethiopia.

## 2. Materials and Methods

### 2.1. Study Area

Wolaita Sodo is located about 390 km south of Addis Ababa. The town Sodo, which is established at the foot of mountain Damota, currently serves as the capital of the Wolaita Zone. The zone is located at the latitude of 8°50°N and a longitude of 37°45°E. Topographically, the area is marked by hilly, flat, steep slopes and gorges and several streams and mountains. The highest mountain is Damota, 2500m above sea level, which is located near Sodo town. The Altitude varies from 1100 to 2950 m.a.s.l. The area experiences a mean annual temperature of about 20 °C. The mean maximum temperature is 26.2 °C and the average monthly minimum temperature is 11.4 °C. The rainfall regimes over much of the area are typically bimodal with the big rainy season extending from June to September and a small rainy season occurring from February to April. The mean annual rainfall of the area ranges from 450 to 1446 mm with the lowest being on low land and the highest on high land. The livestock population in the area is estimated to be 68,900 cattle, 1992 sheep, 382 goats, 121 horses, 131 mules, 488 donkeys, and 55,191 chickens [16].

### 2.2. Study Population

The study population of the current study was nonvaccinated chickens which do not show the clinical signs of the Newcastle disease like torticollis, wing, and leg paralysis, greenish diarrhea, abnormal shaped and shelled eggs, obvious depression, inappetence, increased respiration, swollen heads, and cyanotic combs [17]. The chickens are categorized under three age groups <8 weeks as young, 9–20 weeks as growers, and >20 weeks as adults for layers and from four weeks up to six weeks' age (young) for broilers and based on their purpose as a layer, broiler, and dual. These chickens were reared under an intensive, semi-intensive, and extensive management system by smallholder poultry farm farmers in the Sodo Zuria district.

#### 2.2.1. Inclusion Criteria

Chickens that are apparently (clinically) healthy and with no history of vaccination upon detailed physical and clinical examination and questionnaire survey, respectively, and are managed under a semi-intensive, intensive, and backyards production system were included in the current study.

#### 2.2.2. Exclusion Criteria

All chickens with a history of vaccination, immediate ailment upon questionnaire survey, and immediate physical examination, respectively, and those under four weeks of age were excluded from the study.

### 2.3. Study Design

A cross-sectional study to determine the seroprevalence of the Newcastle disease was conducted in small-scale producer farms in Sodo Zuria district, Wolaita Sodo, southern Ethiopia, from May to July 2021 via collecting blood samples from randomly selected chickens and tested by serological test iELISA [18]. A total of 120 semistructured questionnaires were prepared, and a face-to-face interview was conducted with the higher managers and employees of the farms to assess the associated risk factors of NCD. Then, from the total of 40 farms found in the study district, 30 farms were included for serological study through random selection.

### 2.4. Sample Size and Sample Collection

A simple random sampling technique was employed to select the study population. However, the poultry farms were selected purposively based on the absence of vaccination history and the recent outbreak of Newcastle disease on the farm. Since there was no previous study showing the seroprevalence of the Newcastle disease in the study area, the present study considered 50% expected prevalence, 95% confidence level, and 5% absolute precision or marginal error. Based on these assumptions, the total number of animals to be included in the study was determined using a formula derived by [19]:

(1) $$\begin{array}{c} \frac{n=Z^{2}{\;}^{*}\text{Pexp}\left(1-\text{pexp}\right)}{d^{2}}, \end{array}$$

where *n* = required sample size, *d* = desired absolute precision = 0.05, *Z* = statistic for level of confidence = 1.96^2^, and Pexp = expected prevalence (50%)

Accordingly, the substitution of the value in the above formula gives the required sample size of 384 chickens.

### 2.5. Study Method

#### 2.5.1. Questionnaire Survey

A pretested semistructured questionnaire was prepared and a total of 120 smallholder poultry farm managers and employees of Sodo Zuria district were interviewed to assess associated risk factors of Newcastle disease and to get the history of vaccination and the recent outbreak of the Newcastle disease in the farms. They were briefed on the scope of the study and the confidentiality of all information was provided for them. Questions included the poultry profile (age, sex, breed, and type), management system, housing system, health and hygiene-related practices like vaccination, disinfection, and presence of footbath, health problems and management, owner's extent of knowledge about poultry disease, and the record management of the farm. Before the administration of the questionnaire, oral consent was obtained from the respondents to participate in the interview. The questionnaire was surveyed from May 27 up to June 4, 2021.

#### 2.5.2. Clinical Examination of Chickens

Before sampling, a system-by-system approach of clinical examination of the body parts of chickens was conducted. Then, chickens with no apparent signs of ailment were selected and a blood sample was collected.

#### 2.5.3. Sample Collection and Transportation

A total of 384 blood samples were collected from apparently healthy nonvaccinated chickens (greater than four-week-old) after strict disinfection of Vena ulnaris (wing vein/brachial vein). A 2 ml of blood was drawn using 3 ml syringes with 21-gauge needles. Then the blood was transferred to a labeled plain vacutainer tube and placed in a nearly horizontal position in a cool place to separate serum from the whole blood. Then the serum was extracted within 24 hours into the labeled cryovials and stored at −20 °C until it was processed [20, 21].

#### 2.5.4. Serological Analysis

The collected sera were tested for the presence of antibodies against avian orthoavulavirus 1 (AOaV-1; formerly avian paramyxovirus-1; Paramyxoviridae family) by using a commercially available indirect ELISA kit (ID Screen® Newcastle Nucleoprotein Indirect version 2 kit manufactured by IDvet innovative diagnostics) according to the manufacturer's instructions [22–24]. The test works on the concept of antibodies present in serum samples inhibiting plate-bound nonstructural protein (NSP) antigen. Any antibody that recognizes the antigen in the wells attaches to it and forms an antigen/antibody combination on the well plate surface. The sample was tested in Wolaita Sodo regional veterinary laboratory by iELISA test. For each sample, the sample-to-positive ratio and the antibody titer were calculated to interpret the result as directed by the manufacturer by using its software. Consider the following:

(2) $$\begin{array}{c} S/p=\frac{\text{OD sample}-\text{OD negative control}}{\text{OD positive control}-\text{OD negative control}},\\ \text{Titer}=10^{\log\;10\left(\text{titer}\right)}. \end{array}$$

#### 2.5.5. Data Management and Statistical Analysis

The data collected was entered, filtered, and coded in a Microsoft Excel worksheet and then subjected to statistical analysis using STATA 20 and the result of the study was explained using descriptive statistical methods. Variable such as seroprevalence of Newcastle disease was first described using means and proportions. Bivariate logistic regression was computed to estimate the magnitude of association between risk factors and the disease. Risk factors having a significant association with the disease were further analyzed by multivariate logistic regression analysis using a 95% confidence level (CI). The *p*-value was held at less than 0.05 to define significant differences.

### 2.6. Ethical Consideration and Consent to Participate

Written ethical approval and consent for this study were obtained from the Wolaita Sodo University Research Ethics and Review Committee. Oral consent was also obtained from the farm managers before collecting the blood samples by outlining the purpose of the study and assuring them that it had no an adverse effect. Then, the blood sample were collected from their chickens by adopting strict hygienic measures.

## 3. Results

### 3.1. Assessment Result of Questionnaire Survey

From a total of 40 smallholder poultry farms found in the district, 55% (22/40) farms rear Bovans Browns, 45% (18/40) farms rear Sasso, and 5% (2/40) farms rear Koekoek breeds in addition to Sasso and Bovans Brown breed. Of this, 20% (8/40) farms were intensive, 30% (12/40) farms were semi-intensive, and 50% (20) farms were extensive (Table 1).

There was a footbath at the entry of the poultry house for 15% (6/40) of farms, and an isolation room for 22.5% (9/40) of farms. Out of 40 farms, only 27.5% (11/40) farms have disposal well for dead chickens on their farm, only 37.5% (15/40) farms disinfect the poultry house, and 10% (4/40) of them vaccinate poultry regularly. During the questionary survey, 35% (14/40) keep the hygiene of the poultry house good, 50% (20/40) have medium house hygiene, and 15% (6/40) had poor house hygiene (Table 1).

A total of 120 persons including higher managers and lower employees on a single farm were interviewed, from this 16.67% (20/120) know, 26.67% (32/120) know some, 39.17% (47/120) know little, and 17.5% (21/120) know nothing about poultry disease (Table 1).

### 3.2. Descriptive Statistics of Seroprevalence

**Figure 1:**
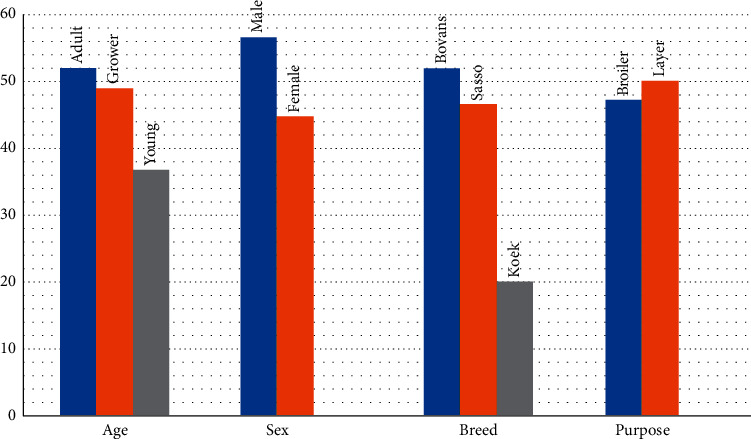
Seroprevalence of NCD in association with animal-related risk factors in Sodo Zuria district.

Seroprevalence of NCD in adults, growers, and young chickens was found to be 51.79% (116/224), 49% (49/100), and 36.67% (22/60), respectively, and 56.59% (73/129) of males and 44.71% (114/225) of female chickens were seropositive. The seroprevalence at breed level was 52% (104/200) in Bovans Brown, 46.55% (81/174) in Sasso, and 20% (2/10) in Koekoek and it was 2.81% and 25.41% in layer than broiler chickens and farms without footbath than those with footbath, respectively (Figure 1).

**Figure 2:**
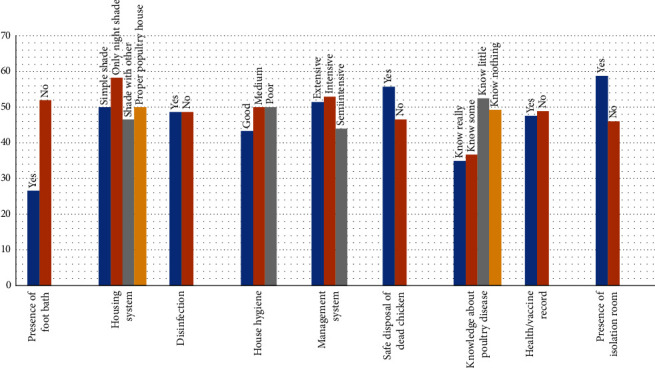
Seroprevalence of NCD in association with management-related risk factors in Sodo Zuria district.

Seroprevalence rate in chickens managed in the simple shade, only nightshade, sharing with other animals and proper poultry house was 50% (64/128), 58.3 (21/36), 46.3% (100/216), and 50% (2/4), respectively. It was 48.68 (37/76) and 48.70 (250/308) on farms with and without disinfection and 51.43% (72/140), 43.95% (69/157), and 52.87% (46/87) in extensive, semi-intensive, and intensive farms, respectively. In farms managed (owned) by people, who know nothing, little, some, and really about poultry disease, the seroprevalence rate was 49.24% (65/132), 52.38% (44/84), 36.67% (22/60), and 35% (7/20), respectively. The absence of health records in farms has little difference (1.34%) in seroprevalence of NCD and it was 58.75% (47/80) and 46.1% (140/304) in farms with or without isolation room (Figure 2).

As the result revealed, the likelihood of female chickens being seropositive for NCD is 0.724 (AOR = 0.724; CI = 0.45–1.16) while keeping male chickens constant. However, the age and breeds of chicken have not shown a statistically significant difference (*p* > 0.05) with the seroprevalence of Newcastle disease (Table 2).

The odds of chickens likely to be infected by the NCD virus in an intensive management system was 0.782 times higher than those chickens kept under a semi-intensive management system (AOR = 0.578; CI = 0.379–1.612) while keeping those chickens raised in an extensive management system constantly. However, the presence of a footbath, housing system, disinfection, and safe disposal of the dead chicken was not shown a statistically significant difference (*p* > 0.05) with the seroprevalence of NCD (Table 3).

## 4. Discussion

The current study demonstrated that 48.7% (*n* = 187/384) of sampled chickens were seropositive for the Newcastle disease. None of the chickens sampled had a history of previous vaccination against ND and were above 4 weeks of age. It is therefore construed that antibodies detected in the small-scale poultry-producing farms in this study were due to natural infection by NDV. However, since the birds were healthy, the reason might be the circulation of the low virulent and pathogenic strain of the virus and NCD is endemic in the study district. This high seroprevalence of NCD implies the maintenance of the virus and the spread of the disease within the study area.

The findings of the current study showed discrepancy with the findings which reported lower seroprevalence of NCD from two local government areas of Ido (11.70%) and Atiba (15.43%) of Nigeria [21], from Alamata district of Tigray region 26.2% (76/214) reported by [25], from Trinidad, West Indias 10% (CI 95%: 4–23%) reported by [26] and the finding of [10] 33.8% (CI: 12.8–38.6%) in Oman. As compared to our study, higher seroprevalences were reported from Batangas, Philippines, by [27] 97.96% (48/49), 79.8% (CI 95%: 70.6–86.9%) in Trinidad, and 80.5% (CI 95%: 70.1–88.5%) in Tobago by [26], from Algeria 82.69% by [28] and from north Gonder (79.6%) by [29].

The findings of the current study were also higher than those of the report of [20] 39.4% (95% confidence interval: 34.6–44.4%) from the Banadir region of Somalia. Also, the result of our study was higher than that of the previous report by [30] which recorded (27.86%) overall seroprevalence in the Agarfa and Sinana Districts of Bale Zone, Ethiopia. This difference in seroprevalence of Newcastle disease between the result of our study and previous reports may be attributed to the type of diagnostic tests employed, the sampling method, study areas, the geographic variation and timing of infection, production system, and breed of poultry tested.

Among anticipated risk factors, sex of the chickens, management system, and safe disposal of dead chickens have shown statistically significant differences (*p* < 0.05) with the seroprevalence of the NCD in the study area. The odds of grower and young chickens being seropositive for NCD were 1.705 and 1.704 (AOR = 1.705, CI = 0.87–3.33; AOR = 1.704, CI = 0.89–3.24), respectively, while keeping adult chicken constant but it was not statistically significant as the finding of [30]. Thus, adults are 1.704 times more susceptible than young to NCD (CI = 0.89–3.24). Growers are 1.705 (CI = 0.87–3.33) times more likely to contract NCD than young. This finding of the current study was in line with the finding of [21] which reported a higher seroprevalence of NCD in adults (7.45%) than growers (6.11%), and the report of [20], showing seroprevalence of NCD was (43.8%) in adults and (19.4%) in growers. This difference may be due to repeated exposure of the adults to the virus. Since all of the chickens sampled were over four weeks of age, the presence of maternal antibodies can be ruled out for such antibodies are known to wan after the age of 3–4 weeks [31].

The findings of this study revealed that females are 0.724 (AOR = 0.724; CI = 0.45–1.16) times more likely to contract NCD than males. The difference in seroprevalence of NCD between the sexes was found to be statistically significant (*x*^2^ = 4.842; *p* = 0.028); thus sex has a significant association with the seroprevalence of NCD. This finding of the current study supports the finding of [25] which reported a statistically significant (*x*^2^ = 4.627; *p* = 0.031) difference between sexes but was inconsonant with the finding of [21, 25] in which seroprevalence of NCD in males (8.24%) was higher than that in females (5.32%). This higher seroprevalence in females may be attributed to their production (egg-laying) stress. The result of this study reveals that higher seropositivity for NCD was seen in Bovans Brown breeds (52%) followed by Sasso breed (46.55%) and then by Koekoek breeds (20%) but the difference was not statistically significant (*p* > 0.05), which agrees with the finding of [24].

The occurrence of NCD in Bovans Brown breeds was 1.839 (AOR = 1.839; CI = 0.11–6.55) higher in Koekoek breeds of chickens. The current study found that the seropositivity of layers (50%) for NCD was higher than the seropositivity of broilers (47.19) and that the likelihood of layers being infected by NCD was 3.59 (AOR = 3.59; CI = 0) times higher than the broilers. But the difference does not show statistical significance (*p* > 0.05), which corroborates the finding of [30] which reported there was a difference among the types of chicken with statistical significance (*x*^2^ = 11.2443; *p* < 0.001). This finding of our study disagrees with the finding of [32] which reported higher seropositivity in broilers than in the layers. The present study reveals that the seropositivity of chickens for NCD has differences according to the housing system, in which higher seroprevalence was seen in chickens reared in the only nightshade (58.3) followed by those reared in the simple shade (50%) and proper poultry house (50%) and then by those sharing house with other animals, but the difference was not statistically significant (*p* > 0.05). Thus, those reared in the only nightshade and shared house with other animals were 1.044 (AOR = 1.044; CI = 0.395–2.758) and 0.762 (AOR = 0.762; CI = 0.41–1.42) times more susceptible to NCD than those in simple shade, respectively. Chickens sampled from houses with and without disinfection have no difference in seroprevalence of NCD 48.7% in both. This may be due to the number of chickens sampled. But the logistic regression of the data showed that the poultry in farms without disinfection was 0.65 (AOR = 0.65; CI = 0.358–1.185) times more likely to contract NCD than in farms that disinfect poultry houses.

The findings of this study demonstrate that the likelihood of chicken reared in intensive and semi-intensive farms being seropositive for NCD was 0.782 (AOR = 0.782; CI = 0.379–1.612) and 0.578 (AOR = 0.578; CI = 0.379–1.612) while keeping those chicken reared in extensive management system constantly. Thus, there was a statistically significant (*p* = 0.0001) association between the management systems and seroprevalence of NCD. This probability may be attributed to the confinement and higher chance of close contact with poultry in intensive and semi-intensive management systems than in extensive ones, which are in line with the previous reports [33, 34]. According to our study, the likelihood of contracting NCD was 0.684 (AOR = 0.684; CI = 0.388–1.209) times higher in poultry kept on farms that do not dispose of dead chickens safely than in those that dispose of them hygienically. This showed a statistically significant (*p* = 0.496) association between the manner of disposal of dead chickens with the occurrence of NCD, which corroborates with the finding of [35, 36].

## 5. Conclusion and Recommendations

The result of the current study demonstrates that NCD was endemic and circulating in the study area with a high 48.7% (*n* = 187/384) prevalence. The sex of chickens, the management system, and the manner of disposal of dead chickens were found to be the contributing risk factors for the occurrence, as well as the endemicity, of the NCD. Therefore, further and detailed investigations including molecular studies have to be carried out on the characteristics of circulating strains and models of transmission for a better understanding of ND epidemiology to develop and implement an evidence-based control program and minimize the economic and social impacts of ND on smallholders' poultry farms in Sodo Zuria district.

## Figures and Tables

**Table 1:** The descriptive statistics questionnaire survey.

Variables		Frequency (%)
Breed of chicken raised	Sasso	45 (18/40)
	Bovans Brown	55 (22/40)
	Koekoek	5 (2/40)

Management system	Intensive	30 (8/40)
	Semi-intensive	30 (12/40)
	Extensive	50 (20)

Presence of footbaths on the farms		15 (6/40)
Presence of isolation room		22.5 (9/40)
Disinfection of poultry house		37.5 (15/40)
Disposing well for a dead chicken		27.5 (11/40)

Poultry house hygiene	Good	35 (14/40)
	Medium	50 (20/40)
	Poor	15 (6/40)

Knowledge of farm owners about poultry disease	Know	16.67 (210)
	Know some	26.67 (32/120)
	Know little	39.17 (47/120)
	Know nothing	17.5 (21/120)

**Table 2:** Summary of univariable and multivariable logistic regression analysis of animal level risk factors associated with NCD in Sodo Zuria district.

Variable	Category	AOR	Chi^2^	*P*-Value	CI (95%)
Age of chickens	Adult	Ref.	4.335	0.114	
	Grower	1.705			0.87–3.33
	Young	1.704			0.89–3.24

Sex of chickens	Male	Ref.	4.842	0.028	
	Female	0.724			0.45–1.16

Breed of chickens	Bovans Brown	Ref.	4.490	0.106	
	Sasso	4.81			0
	Koekoek	0.839			0.11–6.55

Type of chicken	Broiler	Ref.	0.302	0.583	
	Layer	3.59			0

*Note.* AOR is adjusted odds ratio, chi2 is Pearson x2, CI is confidence interval, and Ref. is reference factor.

**Table 3:** Summary of univariable and multivariable logistic regression analysis of farm-level risk factors associated with NCD in Sodo Zuria district.

Variable	Category	AOR	Chi^2^	*P*-value	CI (95%)
Presence of footbath	Yes	Ref.	2.969	0.085	
	No	1.002			0.1595–1.77

Housing system	SS	Ref.	1.926	0.588	
	ONS	1.044			0.395–2.758
	SWO	0.762			0.41–1.42
	PPH	1.952			0

Disinfection	Yes	Ref.	0.0	0.998	
	No	0.65			0.358–1.185

Management system	Extensive		3.84	0.0001	
	Semi-intensive	0.578			0.379–1.612
	Intensive	0.782			0.379–1.612

Safe disposal of the dead chicken	Yes		2.3854	0.496	
	No	0.684			0.388–1.209

SS is simple shade, ONS is only nightshade, SWO is share with other animals, and PPH is proper poultry house.

## Data Availability

The datasets used and analyzed during the current study can be obtained from the corresponding author upon reasonable request.

## Abbreviations

AAvV-1: Avian Avulavirus-1
LoNDV: Low Virulence Newcastle Disease Virus
vNDV: Virulent Newcastle Disease Virus
AOaV-1: Avian Orthoavulavirus-1
iELISA: Indirect ELISA
